# NOS3 gene intron 4 a/b polymorphism is associated with ESRD in autosomal dominant polycystic kidney disease patients

**DOI:** 10.1590/2175-8239-JBN-2021-0089

**Published:** 2022-01-31

**Authors:** Udit Narayan Padhi, Madhubala Mulkalwar, Lakkakula Saikrishna, Henu Kumar Verma, LVKS Bhaskar

**Affiliations:** 1 Guru Ghasidas Vishwavidyalaya, Department of Zoology, Bilaspur, India.; 2 Shri Shankaracharya Institute of Medical Sciences, Junwani, Bhilai, India.; 3 Nellore Municipal Corporation, Department of Public Health, Nellore, India.; 4 Comprehensive Pneumology Center, Institute of Lungs Biology and Disease, Department of Immunopathology, Helmholtz Zentrum, 85764 Neuherberg, Munich, Germany.

**Keywords:** Polycystic Kidney, Autosomal Dominant, Polymorphism, Genetic, Kidney Failure, Chronic, Rim Policístico Autossômico Dominante, Polimorfismo Genético, Falência Renal Crônica

## Abstract

**Introduction:**

Endothelial nitric oxide synthase (eNOS) genes have been implicated in renal hemodynamics as potent regulators of vascular tone and blood pressure. It has been linked to a reduction in plasma nitric oxide levels. Several studies have recently been conducted to investigate the role of *NOS3* gene polymorphisms and end-stage renal disease (ESRD). However, the results are still unclear and the mechanisms are not fully defined. As a result, we conducted a meta-analysis to examine the relationship between *NOS3* gene polymorphism and ESRD in autosomal polycystic kidney disease (ADPKD) patients.

**Methods:**

To assess the relationship between *NOS3* gene polymorphism and ESRD, relevant studies published between September 2002 and December 2020 were retrieved from the PubMed (Medline), EMBASE, Google Scholar, and Web of Science databases. The pooled odds ratio (OR) and 95 % confidence interval (CI) were calculated using a fixed-effect model. To assess the heterogeneity of studies, we used Cochrane’s Q test and the Higgins and Thompson I^2^ statistics.

**Results:**

Our meta-analysis of 13 studies showed that the presence of the two *NOS3* gene polymorphisms significantly increased ESRD risk in ADPKD patients with 4a/b gene polymorphism (aa+ab vs. bb: OR=1.95, 95% CI=1.24-3.09, p=0.004). In addition, no significant association was found between the *NOS3* 894G>T (Glu298Asp) polymorphism and the risk of ESRD in ADPKD patients (GT+TT vs. GG: OR=1.21, 95% CI=0.93-1.58, p=0.157). There was no evidence of publication bias.

**Conclusions:**

The findings of the current meta-analysis suggest that *NOS3* intron 4a/b polymorphism plays a vital role in the increasing risk of ESRD in ADPKD patients.

## Resumo

**Introdução:**

Genes da óxido nítrico sintase endotelial (eNOS) têm sido implicados na hemodinâmica renal como potentes reguladores do tônus vascular e pressão arterial. Tem sido vinculado a uma redução nos níveis plasmáticos de óxido nítrico. Realizou-se recentemente vários estudos para investigar o papel de polimorfismos do gene *NOS3* e doença renal em estágio terminal (DRET). Entretanto, os resultados ainda não são claros e os mecanismos não estão totalmente definidos. Como resultado, realizamos meta-análise para examinar a relação entre polimorfismo do gene *NOS3* e DRET em pacientes com doença renal policística autossômica dominante (DRPAD).

**Métodos:**

Para avaliar a relação entre polimorfismo do gene *NOS3* e DRET, recuperou-se estudos relevantes publicados entre Setembro-2002 e Dezembro-2020 dos bancos de dados PubMed (Medline), EMBASE, Google Scholar, Web of Science. Calculamos odds ratio (OR) e intervalo de confiança (IC) de 95% utilizando modelo de efeitos fixos. Para avaliar a heterogeneidade dos estudos, utilizamos teste Q de Cochrane e estatísticas I^2^ de Higgins e Thompson.

**Resultados:**

Nossa meta-análise de 13 estudos mostrou que a presença dos dois polimorfismos do gene *NOS3* aumentou significativamente o risco de DRET em pacientes com DRPAD com polimorfismo do gene 4a/b (aa+ab vs. bb: OR=1,95; IC 95%=1,24-3,09; p=0,004). Ademais, não encontramos associação significativa entre polimorfismo 894G>T *NOS3* (Glu298Asp) e risco de DRET em pacientes com DRPAD (GT+TT vs. GG: OR=1,21; IC 95%=0,93-1,58; p=0,157). Não houve evidência de viés de publicação.

**Conclusões:**

Achados da meta-análise atual sugerem que o polimorfismo intron 4a/b do *NOS3* desempenha papel vital no aumento do risco de DRET em pacientes com DRPAD.

## Introduction

Renal cysts can have various etiologies, broadly classified into genetic and non-genetic disorders. The most common and widely accepted genetic cause of renal cystic disease in humans is autosomal dominant polycystic kidney disease (ADPKD), autosomal recessive polycystic kidney disease (ARPKD), and juvenile nephronophthisis, for which genes or chromosomal locations have been identified^1^. Among all renal cystic diseases, ADPKD is genetically heterogeneous and affects all racial groups worldwide, associated with liver, cardiovascular, gastrointestinal and genital abnormalities, with an estimated frequency between 1:400 and 1:1,000^2^. ADPKD may be caused by mutations in one of the two genes, namely polycystic kidney disease 1 (*PKD1*), mapped to 16p13.3, and polycystic kidney disease 2 (*PKD2*) gene on chromosome 4q21^3^. The frequency of mutations of *PKD1*gene is much higher and the gene is responsible for 85% of ADPKD cases, while 15% is caused by the *PKD2* gene. Additionally, elderly patients have more cases of *PKD2* mutations than of *PKD1* mutations. Renal disease involves hypertension, urinary tract infections (UTI), hematuria, renal pain, and renal insufficiency, and end-stage renal disease (ESRD) occurs in approximately 50% of ADPKD patients in their late forties^4^.

ESRD is a multifactorial disease and has been shown to be more significant in many aspects of this genetic factor. Previous evidence has proven that impaired nitric oxide synthase 3 (NOS3) contributes to vascular endothelial dysfunction, and blood endothelial cells are suggested to play a vital role in the pathogenesis of ESRD^5^. *NOS3* is a dimeric cellular signaling molecule with significant regulatory activities such as glomerular vasodilatation, which is essential for controlling the glomerular filtration rate (GFR). In this perspective, *NOS3* in ADPKD was considered a possible candidate gene for ESRD^6^. The *NOS3* gene was mapped to a chromosome of 7q36 consisting of 26 exons. Several polymorphic variants have been associated to modified NO synthesis, including promoter-786 T > C, 894 G > T, and intron 4 variable tandem repeats a / b (VNTR) polymorphisms. Among these, the 27-base pair (bp) (VNTR) intron-4 of *NOS3* is known to alter eNOS expression and cause impaired NO synthesis^7^. The genotypes and haplotypes of *NOS3* tagSNPs were not associated with the disease^6^.

Although many researchers examined the association between *NOS3* gene polymorphisms and ESRD in ADPKD, the findings were inconsistent^7-12^. In view of the clinical heterogeneity of ADPKD, this study aimed to quantitatively summarize the association between *NOS3* polymorphisms (894G>T intron 4 VNTR a / b polymorphism) and ESRD risk in ADPKD by conducting a comprehensive meta-analysis of all eligible case-control studies.

## Materials and methods

### Identification of eligibility studies

Articles published between September 2002 to December 2020 on the associations of the *NOS3* gene polymorphism and ESRD were identified. All case-control studies considering the association in ADPKD patients published in English languages were selected and organized according to the PRISMA guidelines^13^.

A comprehensive search was conducted in electronic databases including PubMed (Medline), EMBASE, Google Scholar, and Web of Science with the combination of the following keywords and subheadings: “endothelial nitric oxide synthase”, “eNOS”, “*NOS3*”, “ESRD”, “intron 4 VNTR”, “meta-analysis”, “case-control”, “894G>T (Glu298Asp; rs1799983)”. The last search was carried out on 30 February 2021.

### Inclusion and exclusion criteria

To conduct a more robust meta-analysis, two authors collected data from all relevant articles independently. Our selection criteria were: (1) case-control studies on the association between *NOS3* polymorphisms and risk of ESRD in ADPKD, (2) available full-text articles, (3) written in English, and (4) original data and complete genotype allele count for both the case and control groups were available. Studies were excluded if they (1) had overlapping/duplicate data, (2) had no control group, (3) did not have clear genotype data, and (4) were case reports and review articles. All information about the selection of studies in ADPKD patients with or without ESRD was arranged.

### Statistical analysis

The genotype data of case and control groups were recorded. The comparison group already included a selection, but NOS3 genotypes were not tested for Hardy-Weinberg equilibrium (HWE). The meta-analysis was carried out using the web tool MetaGenyo. For each study, the strength of association between ESRD risk in ADPKD and *NOS3* gene polymorphisms (894G>T and intron 4 VNTR) was assessed by the summary odds ratio (OR) and the 95% confidence interval (CI) in the dominant model. To assess the between-study heterogeneity, we used Q and I^2^ statistics in all studies. To assess the robustness of findings, sensitivity analysis was performed using a “leave-one-out” meta-analysis. Egger’s test and Begg’s funnel plot were used to assess publication bias. All statistical analyses were done using the Comprehensive Meta-analysis software. A P-value of <0.05 was considered statistically significant.

## Results

### Characteristics of the studies

**Figure 1:**
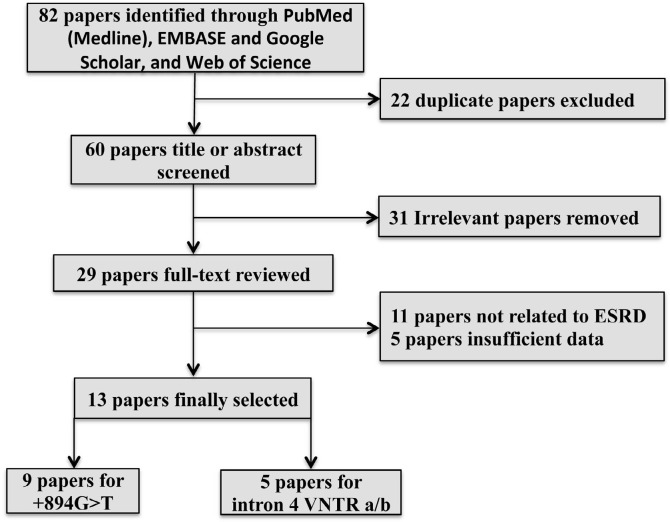
Flow diagram depicting the detailed process of literature search.

Using the aforementioned search strategy, 82 ar­ticles were identified. From these, 22 duplicate and irrelevant articles were excluded. After reading the abstracts and titles, 31 articles that did not assess the association between *NOS3* 894G>T and intron 4a/b polymorphisms and ESRD risk were excluded. Twenty-nine articles were fully reviewed, of which 9 papers with 520 ADPKD patients with ESRD and 563 ADPKD patients without ESRD for the *NOS3* 894G>T polymorphism^8,10-12,14-18^ and 5 papers with 185 ADPKD patients with ESRD and 223 ADPKD patients without ESRD for *NOS3* intron 4a/b polymorphism^7,9,11,19,20^ that had sufficient data were included in the present meta-analysis. The process of selecting papers is depicted in Figure 1. The characteristics of all included studies are listed in Table 1.

**TABLE 1:** Characteristics of the studies on the 894G>T and intron 4 VNTR A/B polymorphisms included in the meta-analysis

894G>T (Glu298Asp) Polymorphism								
Author	Ethnicity	Genotyping	ESRD			No-ESRD		
			GG	GT	TT	GG	GT	TT
Lee et al. (2002)^14^	Asian	PCR-RFLP	20	8	0	65	19	0
Walker et al. (2003)^15^	Caucasian	PCR-RFLP	39	50	7	47	56	16
Reiterová et al. (2004)^16^	Caucasian	PCR-RFLP	37	29	7	61	22	8
Stefanakis et al. (2008)^8^	Caucasian	PCR-RFLP	39	32	9	9	6	5
Dasar et al. (2012)^10^	Caucasian	PCR-RFLP	28	14	0	33	8	1
Ramanathan et al. (2014)^6^	Caucasian	FRET	34	13	1	39	14	1
Pandita et al. (2017)^12^	Asian	ARMS-PCR	27	14	1	63	17	1
Kocyigit et al. (2018)^17^	Caucasian	PCR-RFLP	43	30	5	12	12	6
Malakoutian et al. (2020)^18^	Caucasian	PCR-RFLP	18	10	5	24	16	2
**Intron 4 VNTR a/b**								
			**bb**	**aa+ab**		**bb**	**aa+ab**	
Reiterová et al. (2002)^20^	Caucasian	PCR-RFLP	30	20		72	33	
Merta et al. (2002)^19^	Caucasian	PCR-Electrophoresis	21	9		22	8	
Lamnissou et al. (2004)^9^	Caucasian	PCR- Electrophoresis	10	11		21	7	
Ramanathan et al. (2014)^11^	Asian	PCR- Electrophoresis	31	17		45	9	
Elumalai et al. (2014)^7^	Asian	PCR- Electro-phoresis	25	12		16	0	

### Meta-analysis of NOS3 gene polymorphisms and esrd risk in ADPKD

**Figure 2:**
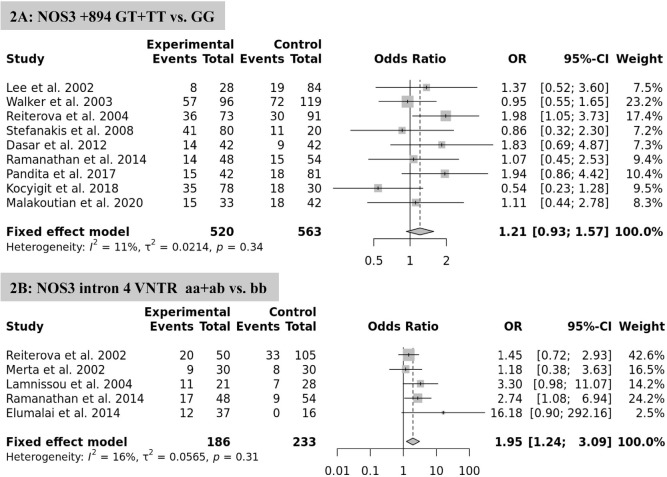
Forest plot of the meta-analysis for the association between NOS3 gene polymorphisms and risk of ESRD in ADPKD patients.

The association between *NOS3* polymorphism variants and ESRD risk in ADPKD was assessed in a dominant model (Figure 2). Overall, the pooled analyses showed that the *eNOS 4a/b* polymorphism is significantly associated with increased risk of ESRD in the fixed-effect model (aa+ab vs. bb: OR=1.95, 95% CI=1.24-3.09, p=0.004) (Figure 2B). However, there was no significant association between *NOS3* 894G>T polymorphism and the risk of ESRD in ADPKD patients (GT+TT vs. GG: OR=1.21, 95% CI=0.93-1.58, p=0.157, fixed-effect model) (Figure 2A).

### Test of heterogeneity, sensitivity, and publication BIAS

**Figure 3:**
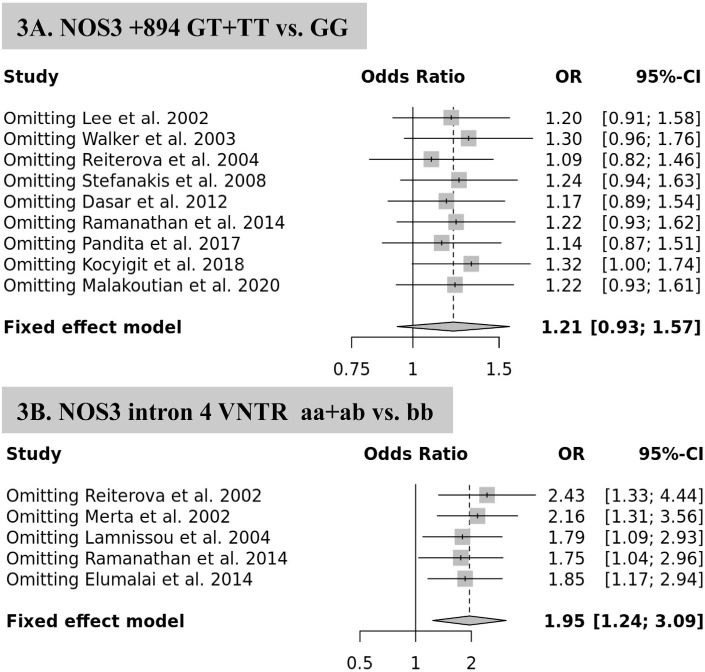
Sensitivity analysis of the association between NOS3 gene polymorphisms and risk of ESRD in ADPKD patients.

The estimated effect sizes for both *NOS3* gene polymorphisms (894G>T: I^2^ = 11.3 %, p-_heterogeneity_ =0.341; and intron 4a/b polymorphism: I^2^ = 15.6 %, p-_heterogeneity_ =0.315) showed significant heterogeneity. Sensitivity analyses were performed by excluding studies one at a time and conducted the analysis after each omission. There were no statistically significant differences in polymorphism data, indicating that the analysis was statistically reliable and consistent (Figure 3A).

**Figure 4:**
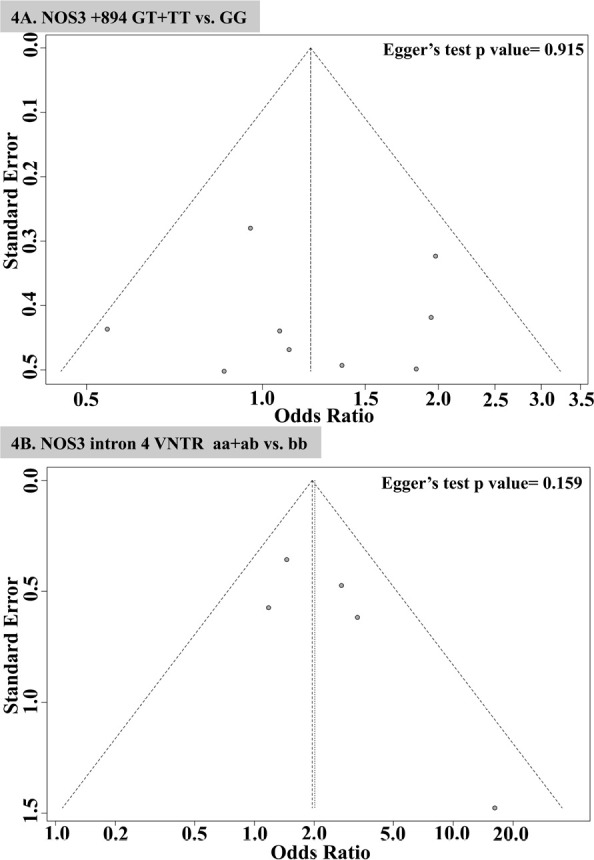
Egger’s funnel plot of publication bias for the NOS3 gene polymorphisms and risk of ESRD in ADPKD patients.

The analysis of the intron 4a/b polymorphism in two studies^19,20^ demonstrated that the pooled ORs increased when each study was omitted (Figure 3B). Begg’s funnel plot and Egger’s test were used to assess the publication bias of the literature. The shape of the funnel plot was asymmetric for both the 894G>T and intron 4a/b polymorphisms. In support of this, the Egger test revealed no evidence of significant publication bias (Figure 4A and 4B). Furthermore, Egger’s linear regression test also revealed no publication bias for studies on the 894G>T (P = 0.915) and intron 4 a/b polymorphisms (P = 0.159).

## Discussion

We included 13 published studies in this meta-analysis that revealed that *NOS3* 4a/b polymorphisms were significantly associated with various vascular complications, which are a cause of ESRD in ADPKD patients. Our study also showed that there is no heterogeneity or publication bias in the included studies. However, the results of the sensitivity analysis in each study group indicated that the pooled OR estimates were not changed quantitatively after each omission. Although the study suggests that nitric oxide may play a role in ADPKD pathophysiology, the *NOS3* 894G>T polymorphism failed to demonstrate an association with susceptibility to ESRD in ADPKD patients. The findings are consistent with a previous meta-analysis that found that the *NOS3* intron 4a/b polymorphism increased the risk of ESRD in ADPKD patients^21^.

The NO synthases (NOS) are a family of enzymes that catalyze the production of nitric oxide (NO) from L-arginine in vascular endothelial cells^22^. It is well known that NO is highly reactive due to its short half-life and potent regulator of vascular tone and hemorheology via the activation of the cyclic guanosine monophosphate (cGMP)-dependent pathway^23^. Besides, it has directly involved in the vascular endothelium, complex cellular interactions, and global inflammation-mediated cell activation^24^. Generally, NO inhibits NaCl absorption along the nephron. Several studies have shown that NOS inhibitors such as nitro-L-arginine and NG-nitro-L-arginine methyl ester (L-NAME) have a tonic influence, especially on medullary circulation^25^. Despite this, renal NO synthesis is involved in the acute and chronic regulation of sodium balance.

Hypertension is the most frequent complication in ADPKD patients, occurring in approximately 60% of the patients. Miyamoto et al. discovered that the 894G>T (Glu298Asp) missense variant was significantly associated with essential hypertension, suggesting a genetic susceptibility for essential hypertension^26^. Another study found that eNOS expression and eGFR were significantly higher in ADPKD patients without hypertension than in those with hypertension. This demonstrates that eNOS gene expression is independently predictive of hypertension in the ADPKD population^27^.

In kidney disease, NO production is reduced by either a decrease in the enzyme substrate (L-arginine) or an increase in the bioavailability of the enzyme inhibitor asymmetric dimethylarginine (ADMA), which in turn reduces NO synthesis via a feedback mechanism^28^. This mechanism has been shown to accelerate the progression of pre-existing kidney disease. Various studies have shown that NO negatively regulates the renin-angiotensin system by inhibiting ACE activity and AT1 receptors^29^. The release of NO by endothelial cells plays a major role in regulating the local hemodynamics and systematic blood pressure^30^. Decreased production of NO plays a major role in the progression of renal disease^28^. A significant decrease in different isoforms of NOS in the cystic epithelium was observed during the growth of a renal cyst in *Han: Sprague-Dawley* (SPRD) polycystic rats^31^. Several lines of evidence suggest that ADPKD is characterized by endothelial dysfunction caused by impaired NO release^32,33^.

Animal models and clinical trials have demonstrated the importance of NOS in polycystic kidney disease^34^. ADPKD patients with the 4a allele progressed to ESRD more slowly in Belgium and France, whereas Hellen’s patients from Greece and Cyprus progressed faster^35,36^. However, some studies suggested that this locus was not linked with ESRD of different etiologies^36,37^. The substitution of aspartic acid for glutamate affects the domain of the oxidase enzyme that serves as a binding site for BH4 and the amino acid L-arginine. The change causes an enzyme variation, making it more susceptible to proteolytic cleavage in position D238-P239. Further, it produces a shorter form of the enzyme, resulting in less NO production^38^. However, the relationship between 894G>T polymorphism and the age of onset of ESRD in ADPKD patients also yielded inconsistent results^15,16,37^. While the-786T>C (rs2070744) polymorphism has a functional effect, it is linked to the replication protein A1 (RPA1), which binds to the NOS3 promoter with high affinity when the C allele is present, resulting in reduced NOS3 transcription^39^.

No significant findings were observed regarding the promoter-786 T>C polymorphism with ESRD progression in patients with type 1 ADPKD^35,40^. Our findings were inconclusive in terms of ethnicity-specific associations between *NOS3* gene polymorphisms and ESRD in ADPKD patients. This is possibly due to differences in the allele frequencies of the *NOS3* gene among populations. Individual studies investigating the link between NOS3 polymorphisms and ESRD complications have also been conducted. Still, these studies may have been imbalanced due to the inability of individual components to identify the desired impact of these polymorphisms.

Certain limitations and biases of the study have to be considered and resutls should be interpreted with caution. Our meta-analysis indicated that the *NOS3* intron 4a/b polymorphism, but not the G894T polymorphism, seems to increase the risk of ESRD in ADPKD patients. More high-quality studies are needed to investigate the complexities of the associations between *NOS3* gene polymorphisms and the therapeutic implications of ESRD in ADPKD patients.
